# Intelligent Algorithms for the Detection of Suspicious Transactions in Payment Data Management Systems Based on LSTM Neural Networks

**DOI:** 10.3390/s25216683

**Published:** 2025-11-01

**Authors:** Abdinabi Mukhamadiyev, Fayzullo Nazarov, Sherzod Yarmatov, Jinsoo Cho

**Affiliations:** 1 Department of Computer Engineering, Gachon University, Sujeong-gu, Seongnam-si 13120, Republic of Korea; mukhamadiyev@gachon.ac.kr; 2 Department of Artificial Intelligence and Information Systems, Samarkand State University, Samarkand 140104, Uzbekistan; fayzulla-samsu@mail.ru (F.N.); sherzod2601@gmail.com (S.Y.)

**Keywords:** payment systems, artificial intelligence, suspicious transaction, data reliability, neural network model, intelligent algorithms, Artificial Bee Colony

## Abstract

Today, a number of works are being carried out all over the world to develop data processing and management systems, as well as to apply artificial intelligence and information technologies in the fields of production, science, education, and healthcare. The optimization of the management of socio-economic process systems, and the management and reliability of databases of the digital payment information-based information systems of enterprises and organizations are relevant. This research work investigates the issue of increasing the reliability of information in information systems working with payment information. The characteristics of ambiguous suspicious transactions in payment systems are analyzed, and based on the analysis, preliminary data preparation stages are carried out for the intelligent detection of ambiguous suspicious transactions. Traditional and neural network models of machine learning for the detection of suspicious transactions in payment information management systems are developed, and a comparative analysis is carried out. Furthermore, to enhance the performance of the core LSTM model, an Artificial Bee Colony (ABC) optimization algorithm was integrated for automated hyperparameter tuning, which improved the model’s accuracy and efficiency in identifying complex fraudulent patterns.

## 1. Introduction

**Figure 1:**
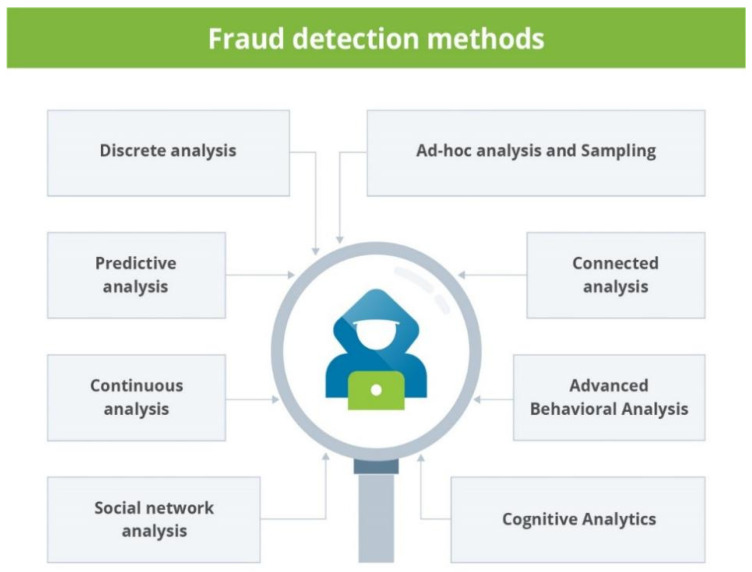
Methods for identifying suspicious transactions.

Currently, payment data is mainly monitored using online systems. Regular monitoring and control of ambiguous suspicious data in information systems developed using distributed methods is required. Several types of ambiguous suspicious data appear in information systems that mainly consist of payment data. The process of monitoring and identifying suspicious transactions in payment systems makes it possible to prevent fraud in these systems. To increase the reliability of data in payment data monitoring systems, the use of distributed ledgers, i.e., blockchain mechanisms, and the use of intelligent distributed mechanisms for rapid processing of data in these systems are effective methods [1,2,3]. However, in order to identify ambiguous suspicious data in such a system, it is necessary to conduct an intellectual analysis of each transaction being executed. To identify suspicious transactions in payment systems, there are several types of features in the transaction data received to be considered. Suspicious transactions in the system are considered to be operations that deviate from the usual transaction behavior of an individual or legal entity, are inconsistent, and which may indicate fraud, money laundering, or other illegal activities. These transactions often have common characteristics that distinguish them from legitimate activity [4,5,6]. There are several methods for identifying unclear suspicious transactions in payment systems. These methods are presented in Figure 1 below.

These methods represent different approaches to data analysis and fraud detection. Each method is used to detect different types of fraud and together they help to increase efficiency [5,7,8]. However, due to the development of different technologies, the development of machine and deep learning neural models for detecting suspicious transactions in payment systems is urgent.

Currently, there are several artificial intelligence-based models and algorithms for the intelligent analysis of structured data. Since existing AI models are often designed for specific tasks and lack generalization, a key research challenge is their continuous improvement to enhance performance and adaptability. In this research, the stages of identifying the main calculated characteristics of suspicious transactions, filling in missing values using normalization, standardization, coding methods, and data preparation were carried out. Based on the analysis of machine learning models to identify suspicious transactions using artificial intelligence, algorithms based on improved ensemble methods of machine learning were developed and experimental results were obtained. Since identifying suspicious transactions using artificial intelligence is a time-series process, scientific research was conducted on neural networks, an algorithm based on LSTM neural network architecture was developed, and optimization stages of this algorithm were carried out.

## 2. Literature Review on the Identification of Suspicious Transactions Based on Artificial Intelligence Models

A significant body of research on suspicious transaction identification has been conducted globally. Notably, scientists such as I.M. Adekunle, P. Ozoh, M. Ahmed, A.N. Mahmood, M.R. Islam, B. Baesens, S. Höppner, Z. Sun, G. Fortino, D. Dablain, B. Krawczyk, and N.V. Chawla are conducting scientific research on the identification of ambiguous suspicious data in systems [9,10,11,12].

A significant line of research focuses on the development of methods, models, and algorithms for detecting fraud in illegal transactions. For instance, Adekunle et al. [1] proposed a fraud detection model, while Al-Momani and Aljawarneh [7] explored the use of deep neural networks for predicting fraudulent transactions.

Researchers such as Akhatov A.R. and Nazarov F.M. are conducting scientific research on identifying suspicious activity based on artificial intelligence models and increasing reliability based on blockchain technologies [4,6,13,14].

Researchers such as Khosravi S., Kargari M., Teimourpour B., Eshghi A., Aliabdi A., Kute D. V., Pradhan B., Shukla N., Alamri A., Liu X., and Zhang P. have conducted research on the use of supervised machine learning methods to detect fraud in the banking transaction network, deep learning and explainable artificial intelligence techniques used in money laundering detection, and statistical scanning models for detecting suspicious transactions to combat money laundering in financial institutions [5,8,15].

Other studies have addressed the policy and advisory aspects of fraud mitigation. For example, Ridwan et al. [16] investigated cashless policy strategies to minimize fraud in the government sector, and Rose et al. [17] developed a legal advisory tool for credit card fraud using machine learning.

As a result of the analysis of the above scientific research works, it was determined that this research work is relevant, since the issues of developing universal intelligent algorithms for detecting suspicious transactions in payment data management systems and the development and optimization of ensemble methods of machine learning and LSTM neural network algorithms for intelligent analysis of time-series datasets have not been fully resolved.

## 3. Methodology

### 3.1. Characteristics of Unclear Suspicious Transactions

Suspicious transactions are transactions that deviate from the normal transactional behavior of an individual or legal entity and may indicate fraud, money laundering, or other illegal activities. These transactions often have common characteristics and circumstances that distinguish them from legitimate activity. Examples of situations that may indicate unclear suspicious transactions include the following:

➢ Unusually large transactions. Transactions that are significantly larger than the customer’s usual spending pattern.
➢ Multiple small transactions. A series of small transactions made over a short period of time, transactions that may use hidden parameters to avoid detection limits.
➢ Geographic anomalies. Transactions originating from locations where the customer has never conducted business before.
➢ High-risk countries. Transactions involving countries known for fraud or money laundering activities.
➢ Unusual timing. Transactions made at unusual times for the customer.
➢ Sudden changes in transactions. A sudden increase in transaction frequency or volume without a clear explanation.
➢ Transactions with unusual characteristics, such as transfers between multiple accounts without any apparent reason.

If the above-mentioned situations occur in the data contained in the payment systems, it is necessary to include this data in the list of uncertain suspicious transactions and analyze it. It is not easy to check the data flowing through the system for suspicious transactions, and there are several problems in identifying suspicious transactions. Detecting suspicious transactions involves overcoming several key challenges:

➢ Data volume and velocity. The sheer volume of transactions processed daily and the speed at which they occur can slow down traditional monitoring systems.
➢ False positives. Legitimate transactions can be falsely flagged as suspicious, leading to customer dissatisfaction and unnecessary investigation costs.
➢ False negatives. Undetected fraudulent transactions can result in significant financial losses.
➢ Evolving fraud tactics. Fraudsters are constantly developing new ways to circumvent detection systems, making the constant update and adaptation of monitoring algorithms a requirement.
➢ Data quality. Incomplete, inaccurate, or inconsistent data can hinder the effectiveness of detection systems.
➢ Regulatory compliance. Ensuring that detection systems comply with various legal and regulatory requirements can be complex and resource-intensive.
➢ Balancing security and customer experience: striking the right balance between reliable fraud detection and a seamless customer experience is critical to maintaining customer trust.

There are several methods for detecting ambiguous suspicious data in systems, and the rapid development of information technology has led to a sharp increase in risks in these systems [15]. Therefore, it is necessary to develop intelligent approaches to detecting ambiguous suspicious data in systems. Intelligent approaches to detecting ambiguous suspicious data based on machine learning are several times more effective than traditional methods.

### 3.2. Preparing Initial Data for Intelligent Detection of Unclear Suspicious Transactions

In developing an effective machine learning-based detection system for suspicious transactions, it is important to collect data from various sources. The quality, diversity, and completeness of the data significantly affect the accuracy and reliability of the system.

The experimental dataset used in this study was collected from partner financial organizations within the Samarkand region. Due to the highly sensitive and confidential nature of financial transactions, the dataset is not publicly available and is used under strict non-disclosure agreements to ensure data privacy and security.

The final synthesized dataset consisted of approximately [INSERT NUMBER, e.g., 100,000] transaction records, with a fraud rate of approximately [INSERT PERCENTAGE, e.g., 1.5%], reflecting the typical imbalance found in real-world financial data.

**Figure 2:**
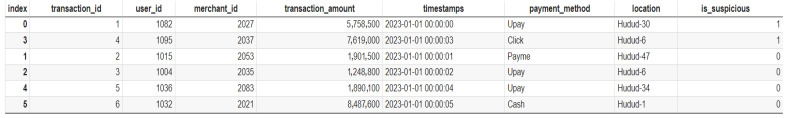
Experimental dataset.

The experimental dataset used in the study to develop the machine learning-based suspicious transaction detection system is illustrated in Figure 2.

The features extracted from the dataset are as follows:

➢ *transaction_id*: An identifier for each transaction.
➢ *user_id*: An identifier for each user. This can help track user-specific characteristics.
➢ *merchant_id:* An identifier for each system employee. This can help track the unique characteristics of an employee.
➢ *transaction_amount:* The amount of money involved in the transaction.
➢ *timestamps*. The date and time the transaction occurred. This can be used to analyze the time-based signatures of transactions.
➢ *payment_method*: The method used for the transaction (e.g., credit card, debit card, wire transfer, PayPal). Different methods may have different levels of risk.
➢ *Location*: The geographic location where the transaction occurred. Unusual locations may be suspicious to the user.
➢ *ip_address*: The IP address from which the transaction was made. This can be used to detect anomalies in network activity.
➢ *is_suspicious*: A binary label indicating whether the transaction is suspicious (1) or not (0). This is the target variable for the machine learning model.

*Preparing the dataset for machine learning*. Preparing the dataset for machine learning for a machine learning-based suspicious transaction-detection system involves several steps. This process is performed in the following steps:

1. Data Cleaning:

*Handling Missing Values.* Missing values are filled in using median values.

*Removing Duplicates.* All duplicate rows, defined as entries with identical values across all features, were removed from the dataset to prevent model bias and ensure data quality.

2. Error Correction and Outlier Handling.

Outliers in numerical features were identified and corrected using the Z-score method. For each feature, data points with a Z-score greater than a predefined threshold of 3 were considered outliers. These outlier values were replaced by the median value of the respective features to maintain data integrity without being skewed by extreme values.

**Figure 3:**
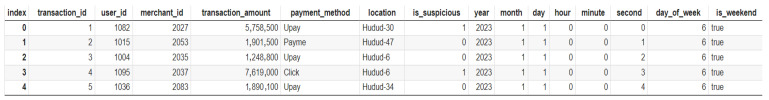
Processed dataset.

3. Feature Engineering:

In the dataset shown in Figure 2, the timestamps, payment method, and location properties are arranged as text data. Since the timestamps property represents time, it needs to be processed and converted into a format that can be used in the model [18,19]. Typically, this involves extracting useful components from the time property, such as year, month, day, hour, minute, second, day of the week, etc. After these components are extracted, the overall view of the dataset is depicted in Figure 3.

### 3.3. Characteristics of Unclear Suspicious Transactions

Machine learning technology is one of the most effective tools for detecting suspicious transactions in payment data monitoring systems. Traditional rule-based systems work according to predefined rules and can make mistakes in detecting newly developed fraud methods. Machine learning, on the other hand, provides the ability to identify complex and ambiguous relationships between transactions with the ability to self-learn.

The following machine learning algorithms can be used to detect suspicious transactions in payment data monitoring systems:

➢ **Logistic Regression.** A simple and effective algorithm that uses linear regression to detect fraud.
➢ **K-Nearest Neighbors (KNNs).** The KNN algorithm classifies transactions based on the dataset to which they are close.
➢ **Basis Vectorization.** A classification algorithm that works on linear and nonlinear data and helps to distinguish suspicious and normal transactions.
➢ **Decision Trees.** This algorithm analyzes transactions and classifies them in a tree structure based on whether they are likely to be suspicious or normal.
➢ **Random Forest.** Used in classification and regression tasks using multiple decision trees as an ensemble. It is effective for fraud detection.
➢ **Gradient Boosting.** A powerful classification method that is also used to detect payment fraud.
➢ **XGBoost.** An updated version of Gradient Boosting, it is a highly efficient algorithm for fraud detection.
➢ **Recurrent Neural Networks.** Used to detect payment sequences, helping to track how fraud manifests itself over time.
➢ **Naive Bayes.** A simple algorithm based on random assumptions, used to detect suspicious transactions.
➢ **k-Means Clustering.** This algorithm is used as a clustering method to detect suspicious transactions, separating them from groups that may appear as anomalies. These algorithms help to strengthen fraud detection in transaction monitoring systems and provide more accurate results. Several algorithms are analyzed in the study.

**Logistic Regression.** This algorithm is a probabilistic classification model, and the main task of the model is to obtain a binary result (e.g., 0 or 1) based on the input features. In this case, a logistic regression model is used to classify suspicious transactions as (1) and normal transactions as (0).

***Mathematical expression of the model.*** In Logistic Regression, the output ${\hat{y}}_{i}$ represents the probability that each transaction is suspicious. This probability is calculated using the input features $X_{i}$ and weights w and the bias b. The modeling rule is as follows:

$${\hat{y}}_{i}=P(y_{i}=1|X_{i})=\sigma ({\bar{w}}^{T}X_{i}+b)$$

where ${\hat{y}}_{i}$ is the probability of a transaction being suspicious, $\bar{w}$ is the weight vector ($w_{1},w_{2},\ldots ,w_{n}$), $X_{i}$ is the input feature vector for transaction ($x_{1},x_{2},\ldots ,x_{n}$), and b is the bias (offset) value.

***Sigmoid function.*** The sigmoid function is the main element of this model, and it is used in logistic regression to approximate the output to two different values (0 or 1). The sigmoid function is expressed as follows:

$$\sigma (z)=\frac{1}{1+e^{-z}}$$

where $z={\bar{w}}^{T}X_{i}+b=w_{1}x_{1}+w_{2}x_{2}+\ldots +w_{n}x_{n}+b$ is the combination of weights and input features.

Algorithms for detecting suspicious transactions in payment data monitoring systems based on Logistic Regression and several other machine learning algorithms have been developed. The overall results are presented in Table 1.

Although the study obtained preliminary results on the detection of suspicious transactions in payment data management systems using traditional machine learning models (Logistic Regression, K-Nearest Neighbors (KNNs), XGBoost, Random Forest), it was found that there are some problems and limitations in these approaches:

It was found that in classic models such as Logistic Regression or KNN, it is very important to identify features well. However, analyzing complex dependencies, ambiguous patterns, or hidden relationships between features is difficult [20,21].

Payment systems constantly generate very large amounts of data. It was found that methods such as KNN can run very slowly on large amounts of data, and Logistic Regression or Random Forest can sometimes have difficulty in representing complex nonlinear relationships well.

Fraud and suspicious transaction patterns change frequently. It was found that even powerful classifiers such as XGBoost and Random Forest sometimes cannot fully represent hidden patterns in complex, multi-dimensional data.

In these classical models, hyperparameter optimization (Grid Search, Random Search) required a lot of time and resources. It was also found that sometimes the correct choice of model parameters makes it difficult to find a balance between overfitting and underfitting [22,23,24].

Due to these limitations, the need to use artificial neural networks arises in the next stage of research. Neural networks (Deep Learning) offer the following advantages:

- Architectures such as DNN, LSTM, and CNN can independently identify complex and hidden features from large amounts of raw data. This saves researchers from the difficulties of the feature engineering stage.
- Neural networks are suitable for representing very complex, nonlinear relationships and can “learn” even more difficult dependencies than traditional models.
- The ability to combine different large-scale types of data (text, numbers, geo-data, time series) and train in the same format. This provides a comprehensive analysis of suspicious transactions.
- Since fraud has changing patterns, neural networks can learn these new patterns over time, adapt, and provide more reliable forecasts.

In conclusion, although the Logistic Regression, KNN, XGBoost, and Random Forest models used in the first stage provided preliminary results, their limitations in fully representing complex and dynamic fraud patterns necessitated the use of neural networks in the next stage of research [25,26,27]. This will improve the quality of the model, identify suspicious transactions with higher accuracy, and increase the adaptability of the system.

### 3.4. Identification of Suspicious Transactions Based on a Neural Network Model of Artificial Intelligence

In payment data monitoring systems, neural networks can be used to detect suspicious transactions. Extensive applications for anomaly detection are essential for predicting the complexity of these datasets.

**Recurrent Neural Networks.** Training Recurrent Neural Networks (RNNs) to detect suspicious transactions in payment data monitoring systems involves several stages.

*RNN architecture*. RNN analyzes time-series data and stores hidden states at each time step. Where $h_{t}$ represents the hidden state at each time step and $X_{t}$ represents the input feature vector at each time step, the hidden states $h_{t}$ are updated at each time step as follows:

$$h_{t}=f(W_{h}h_{t-1}+W_{x}X_{t}+b_{h})$$

where $W_{h}$ is the weight matrix for the hidden state at the previous time step, $W_{x}$ is the weight matrix for the input at the current time step, and $b_{h}$ is the bias (offset) value. Usually, f(⋅) is a nonlinear activation function such as tanh or ReLU.

Output Layer. The output ${\hat{y}}_{t}$ at each time step is calculated as follows:

$${\hat{y}}_{t}=\sigma (W_{y}h_{t}+b_{y})$$

where $W_{y}$ is the weight matrix that transfers the hidden state to the output, $b_{y}$ is the output bias, and σ(⋅) is the sigmoid activation function (for binomial classification).

Loss Function. The loss function for binomial classification is usually binary cross-entropy. The loss, $L_{t}$, for a single transaction, t, is calculated as

$$L_{t}=-\left(y_{t}\log({\hat{y}}_{t})+(1-y_{t})\log(1-{\hat{y}}_{t})\right)$$

where $y_{t}$ is the real label (which indicates whether the transaction is suspicious or not) and ${\hat{y}}_{t}$ is the probability calculated by RNN.

*Backpropagation over time*. During the training process, the overall loss is minimized using gradient descent. At each time step, the loss is backpropagated and used to update the network weights.

Training stages. Based on the above information, the training stages of RNN were expressed as the following steps:

1. The weights are initialized with initial values of $W_{h}$, $W_{x}$, $W_{y}$, and bias $b_{h}$, $b_{y}$.
2. For each training line,
  - The input sequence X is passed through the RNN.
  - At each time step, $h_{t}$ hidden states and ${\hat{y}}_{t}$ outputs are computed.
  - The loss is computed using binary cross-entropy.
  - The loss is propagated back and the weights are updated using gradient descent.
3. This process is repeated until the network reaches convergence, or the specified number of epochs is reached [24,25,26,27].

The above processes illustrate the process of training an RNN model to detect suspicious transactions against transaction data.

***LSTM networks*.** LSTM networks, a type of RNN, are well suited for sequential data due to their ability to capture temporal dependencies and long-term patterns. In the context of transactional data, LSTMs can model the sequence of transactions made by a user over time to detect anomalies or suspicious activity.

***LSTM architecture.*** For each time step t, $x_{t}$ input vectors, $h_{t-1}$ previous hidden states, and $C_{t-1}$ previous cell states are calculated.

Input Gate ($i_{t}$) controls how much new information enters the cell state.

$$i_{t}=\sigma (W_{i}[h_{t-1},x_{t}]+b_{i})$$

Forget Gate ($f_{t}$) determines how much of the previous cell state to preserve.

$$f_{t}=\sigma (W_{f}[h_{t-1},x_{t}]+b_{f})$$

Output Gate ($o_{t}$) controls how much of the cell state is affected by the hidden state.

$$o_{t}=\sigma (W_{o}[h_{t-1},x_{t}]+b_{o})$$

Cell Candidate (${\tilde{C}}_{t}$)

$${\tilde{C}}_{t}=\tanh(W_{C}[h_{t-1},x_{t}]+b_{C})$$

Cell State Update ($C_{t}$) updates the cell state by combining the previous state and the new candidate values.

$$C_{t}=f_{t}*C_{t-1}+i_{i}*{\tilde{C}}_{t}$$

Hidden State $h_{t}$ produces output for the current time step.

$$h_{t}=o_{t}*\tanh(C_{t})$$

where σ is the sigmoid activation function, tanh is the hyperbolic tangent function, and * denotes element-wise multiplication.

After processing the input sequence through the LSTM layers, the final hidden state $h_{T}$ (where T is the last time step) is typically passed through a dense (fully connected) layer with a sigmoid activation function for binary classification (suspect or not).

$$\hat{y}=\sigma (W_{y}h_{T}+b_{y})$$

where $W_{y}$ and $b_{y}$ are the output layer weights and biases and $\hat{y}$ is the estimated probability that the transaction is suspicious.

***Loss Function.*** The loss function in an LSTM network is used to measure the difference between the model’s predictions and the actual values. During training, the model tries to minimize this loss, adjusting its parameters to increase the accuracy of its predictions. For binary classification, the Binary Cross-Entropy (BCE) loss function is usually used.

$$L=-\frac{1}{m}\sum_{k=1}^{m}\left[y_{k}\log({\hat{y}}_{k})+(1-y_{k})\log(1-{\hat{y}}_{k})\right]$$

where m is the number of teaching samples, $y_{k}$ is the true label for the kth sample (0 or 1), and ${\hat{y}}_{k}$ is the predicted probability for the kth sample.

The optimization and training process for each sequence in the training data is as follows:

Forward Pass of the LSTM network. The initial state for $h_{0}$ and $C_{0}$ is entered. LSTM tarmog‘ini oldinga o‘tishi (Forward Pass).

Each time, in steps t=1 to T, the following process is repeated:

- using the above equations, the values of $i_{t}$, $f_{t}$, $o_{t}$, ${\tilde{C}}_{t}$, and $C_{t}$ are calculated;
- the final output value $\hat{y}$ is printed.

Rewinding an LSTM network through time. Over time, the LSTM network calculates the loss gradient, L, with respect to the model parameters (weights and bias). This includes the following:

1. Calculate the loss gradient with respect to $\hat{y}$:
  $$\frac{\partial L}{\partial {\hat{y}}_{k}}=-\frac{y_{k}}{{\hat{y}}_{k}}+\frac{1-y_{k}}{1-{\hat{y}}_{k}}$$
2. To obtain gradients with respect to parameters $W_{y}$ and $b_{y}$, this gradient is fed back through the output layer.
  $$\frac{\partial L}{\partial W_{y}}=\frac{\partial L}{\partial \hat{y}}h_{T}^{T},\;\frac{\partial L}{\partial b_{y}}=\frac{\partial L}{\partial \hat{y}}$$
3. For each t=T to t=1, the gradients of $W_{i}$, $W_{f}$, $W_{o}$, and $W_{C}$ and their corresponding $b_{i}$, $b_{f}$, $b_{o}$, and $b_{C}$ biases are calculated.

**Figure 4:**
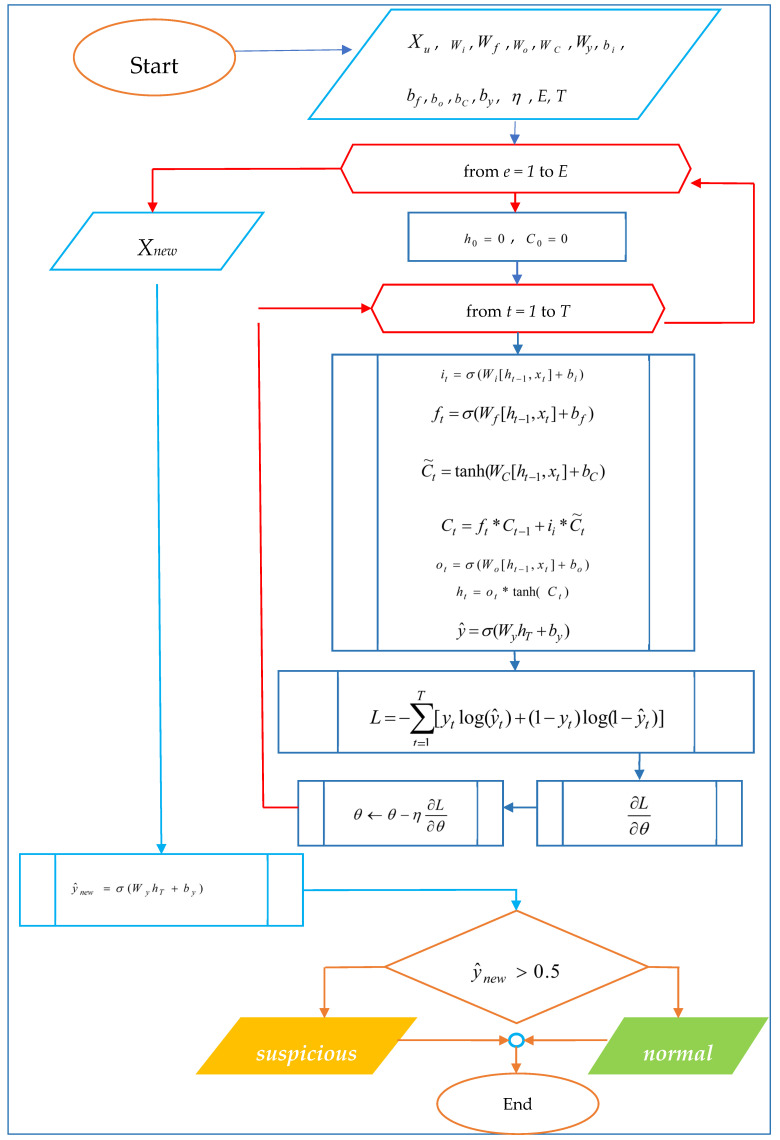
An algorithm based on an LSTM network for identifying ambiguous suspicious data.

The developed model is evaluated using indicators such as Accuracy, Precision and Recall. The block diagram of the mathematical algorithm of the LSTM network for detecting suspicious transactions is shown in Figure 4 below.

**Experimental Results and Discussion.** In payment data monitoring systems, algorithms for detecting ambiguous suspicious transactions based on LSTM networks were developed based on an experimental dataset, and hyperparameters were tuned. The prediction results of the LSTM algorithm are presented in Table 2.

**Figure 5:**
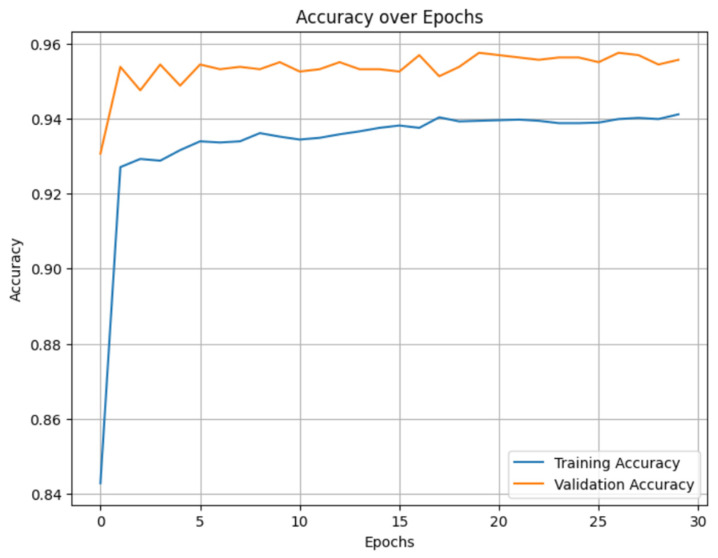
The change in the value of the loss function at each iteration during LSTM training.

The change in the value of the loss function at each iteration during the training of neural networks to detect ambiguous suspicious transactions is represented in Figure 5.

Monitoring the value of the loss function when training neural networks is important for understanding the behavior of the model. If the value of the loss function decreases with an increasing number of epochs, it indicates that the model training process is being carried out well.

As a result, an algorithm developed based on the LSTM neural network makes it possible to identify ambiguous suspicious data in the system and suspicious transactions in payment systems.

**Results analysis.** According to the results of the study on the detection of suspicious transactions in payment data management systems, although the classical machine learning methods used at the initial stage (Logistic Regression, K-Nearest Neighbors, XGBoost, Random Forest) achieved certain results based on specific data and basic features, they encountered limitations in fully representing complex and changing patterns.

**Figure 6:**
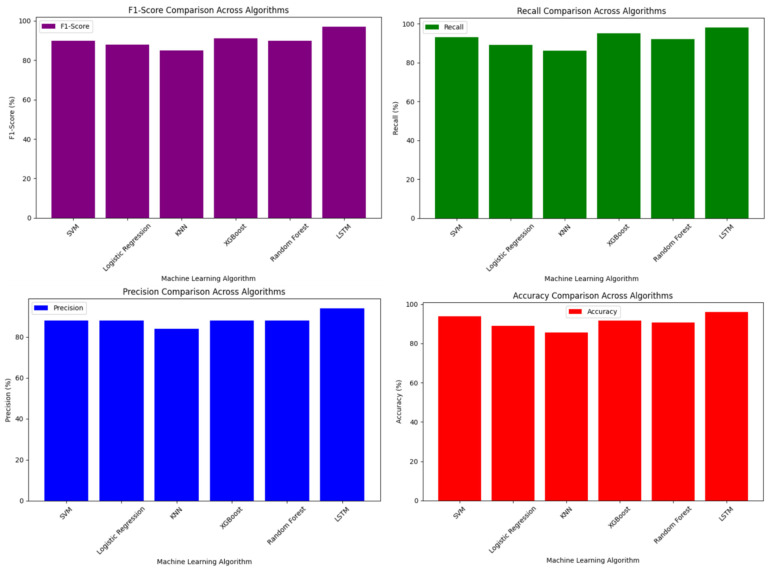
Comparative analysis of the results of the algorithms.

At the next stage, the use of neural networks, in particular the LSTM (Long Short-Term Memory) architecture, made it possible to analyze organizational and time-dependent patterns in more depth. LSTM networks were able to better learn time sequences, dynamic changes, and hidden relationships, and provide more advanced predictions. As a result, the LSTM model provided more accurate and reliable results compared to previous classical models. A comparative analysis of the results of these algorithms is presented in Figure 6.

Thus, the use of neural networks, in particular the introduction of the LSTM model, has significantly improved the quality of suspicious transaction detection. This creates significant opportunities for effective risk management in real time and improving payment security.

In the subsequent section of this research, a methodology for enhancing the previously developed LSTM model is presented. The standard LSTM model showed a big improvement over traditional machine learning methods, but its effectiveness depends greatly on how well its hyperparameters, like the number of hidden layers, learning rate, and batch size, are set. Adjusting these settings by hand can take a long time, not work well, and not have a systematic search strategy. To overcome this limitation and achieve complete automation of the model optimization process, we present a hybrid approach that combines the ABC algorithm with the LSTM architecture. The ABC algorithm is used for automated hyperparameter optimization because it can search the whole space quickly and easily handle difficult, nonlinear optimization problems. By systematically finding the best LSTM configuration, this integration aims to improve the model’s ability to identify complex and changing fraudulent patterns. This will make detection more accurate, robust, and efficient in real-world payment monitoring situations.

***LSTM Neural Network Improvement Method.*** Since the process of detecting suspicious transactions is a time-series problem, it is appropriate to use LSTM neural networks. The use of evolutionary algorithms in determining the hyperparameters of the LSTM neural network is a new approach. The issue of integrating the ABC method to adjust the hyperparameters of the LSTM neural network has been solved. The LSTM neuron network has *t* as the time step for $x_{t}=[x_{1},x_{2},\ldots ,x_{t}]$, which is the entry of a vector, below are listed $h_{t-1}$ as earlier in the hidden condition, and $C_{t-1}$ is the previous condition of the cells.

$$i_{t}=\sigma (W_{i}[h_{t-1},x_{t}]+b_{i}),\;f_{t}=\sigma (W_{f}[h_{t-1},x_{t}]+b_{f}),\;o_{t}=\sigma (W_{o}[h_{t-1},x_{t}]+b_{o}),\;{\tilde{C}}_{t}=\tanh(W_{C}[h_{t-1},x_{t}]+b_{C}),$$

$$C_{t}=f_{t}*C_{t-1}+i_{i}*{\tilde{C}}_{t},\;h_{t}=o_{t}*\tanh(C_{t})$$

LSTM ’s last hidden condition $h_{T}$ (where *T* is the last time/stage) for binary classification is usually $\hat{y}=\sigma (W_{y}h_{T}+b_{y})$, active through sigma.

$$L=-\frac{1}{m}\sum_{k=1}^{m}\left[y_{k}\log({\hat{y}}_{k})+(1-y_{k})\log(1-{\hat{y}}_{k})\right].$$

is the method applied for minimizing the loss function of ABC. The initial P indicates the populations and N consists of bees, and each bee random initial solution S_i_ is given by the value

$$P=\{S_{1},S_{2},\ldots ,S_{N}\}$$

Populations of each $S_{i}$ value, $h_{i}\in [h_{1};h_{n}]$ which is the secret and the layering numbers, and the $\eta_{i}\in [\eta_{1};\eta_{n}]$ learning rate value means can be characterized by (1).

(1) $$S_{i}=(h_{i},\eta_{i})$$

Thus $h_{i}=(h_{1},h_{2}\ldots ,h_{k})$ is the hidden layering, $h_{i}\in [10;200]$ is the *k*–layers of the number, and $\eta_{i}\in [0.00001;0.1]$ is the learning rate parameter for the initial values given. A neighboring solution as a working bee for $S_{i}^{\prime }$ is to obtain the territory of the value $S_{i}$ from minor modifications, with the creation of (2):

(2) $$S_{i}^{\prime }=S_{i}+\varphi_{ij}(S_{i}-S_{d})$$

Thus, the $S_{d}$ in populations randomly selected as $S_{i}$ in the above solution is (d≠i), $\varphi_{ij}\in [-1;1]$, which are random parameters. Every $S_{i}^{\prime }$ solution to $f(S_{i}^{\prime })$, the loss function, is assessed by (3).

(3) $$f(S_{i}^{i})=-\frac{1}{m}\sum_{k=1}^{m}\left[y_{k}\log({\hat{y}}_{k})+(1-y_{k})\log(1-{\hat{y}}_{k})\right]$$

Thus, a ${\hat{y}}_{j}$–LSTM with $S_{i}^{\prime }$ parameters will reach this result.

Good solution selection and the update ($f(S_{i}^{\prime })$) of the original solution with the price of the good is represented by $f(S_{i}^{\prime })<f(S_{i})$, which is the updated solution.

$$S_{i}=S_{i}^{\prime },\;f(S_{i})=f(S_{i}^{\prime }),\;S_{i}=\left\{\begin{array}{l} S_{i}^{\prime },\;agar\;f(S_{i}^{\prime })<f(S_{i})\\ S_{i},\;aks\;holda \end{array}\right.$$

Finder bee populations will replace the solutions having the worst price. However, if the full iteration is not improved without $S_{i}=(h,\eta )$, the random value, it will be recreated on this basis. In the end, the iteration with the best $S=\arg\underset{S_{i}\in P}{\min}f(S_{i})$ solution *S* is selected. New $X_{yangi}$ for the series ${\hat{y}}_{yangi}\sigma (W_{y}h_{T}+b_{y})$ is predicted. If it is suspected that the transaction ${\hat{y}}_{new}>0.5$ is carried out necessarily, this not a decision that is otherwise questionable.

$${\hat{y}}_{new}=\left\{\begin{array}{l} 1\;\sigma (W_{y}h_{T}+b_{y})>0.5\\ 0\;else \end{array}\right.$$

As a result, the minimum value of b in the architecture of the neuron network loss function of the number of hidden layers is determined by speed, the best values in teaching, developing a network of neuron b, and the result of bee colonies through the improvement of the method of (3), which is represented in Table 3.

LSTM neuron network models developed on the basis of this method, which returns the best results of the hyperparameters found, will create opportunities to increase the speed and accuracy of solution finding.

## 4. Conclusions

This research work examined the process of developing intelligent algorithms for detecting suspicious transactions in payment data management systems. The characteristics of suspicious and ambiguous transactions were analyzed. Based on the analysis, the initial data preparation stages were carried out for the intelligent detection of ambiguous and suspicious transactions. A neural network model using artificial intelligence was developed to detect suspicious transactions in payment data management systems. An intelligent algorithm for detecting suspicious transactions based on the neural network model was created, experimental results were obtained, and its advantages over other traditional machine learning models were analyzed. Moreover, the application of the ABC algorithm for optimizing LSTM hyperparameters demonstrated a significant enhancement in detection performance, achieving higher accuracy and robustness in identifying sophisticated suspicious transaction patterns compared to the baseline LSTM model.

## Figures and Tables

**Table 1:** Results of machine learning algorithms used to detect suspicious transactions.

№	Machine Learning Algorithm	Methods for Evaluating an Algorithm (%)			
		Accuracy	Precision	Recall	F1-Score
1	SVM	93.75	88	93	90
2	Logistic Regression	89	88	89	88
3	KNN	85.65	84	86	85
4	XGBoost	91.65	88	95	91
5	Random Forest	90.6	88	92	90

**Table 2:** Prediction results of the LSTM algorithm.

LSTM algorithm	Parameters	Evaluation Methods			
		Accuracy	Precision	Recall	F1-Score
	Epochs = 30	93%	89%	95%	92%
	Batch size = 32				
	$h^{\left(1\right)}=50$				
	$h^{\left(2\right)}=50$				
	Activation function—sigmoid				

**Table 3:** Results of the method for improving LSTM network hyperparameters via the ABC algorithm for detecting suspicious data.

Hybrid Method (LSTM + AKA)	Accuracy	Precision	Recall	F1-Score,
	95%	92%	97%	94%
Parameters	Epochs = 5 when the bat size = 32	$h^{\left(1\right)}=131$ n_bees = 10 n_iterations = 50	Akvitatsiya-sigmoid	η=0.02

## Data Availability

The data presented in this study are available on request from the corresponding author. (The data are not publicly available due to institutional policy and ongoing research confidentiality.)
